# Supplementary material for: The carbon footprint of surgical operations: 2023–2025 systematic review update
Source: PLoS One. 2026 May 18;21(5):e0349415. doi: 10.1371/journal.pone.0349415 (PMC13183196; doi:10.1371/journal.pone.0349415)
Supplement: S2 File — (DOCX) [file pone.0349415.s002.docx]

**Search strategy**

("environmental impact" OR "environmental sustainability" OR "ecological footprint"

OR "carbon footprint" OR "environmental burden" OR "sustainable healthcare"

OR "environmental effects" OR "environmental consequences"

OR "environmental implications" OR "environmental performance"

OR "climate impact" OR "greenhouse gas emissions" OR "GHG emissions" OR “eco-audit”)

AND

("healthcare sector" OR "medical sector" OR "hospital" OR "clinical setting"

OR "healthcare facilities" OR "medical facilities" OR "hospital waste"

OR "medical waste" OR "clinical waste" OR "healthcare services"

OR "healthcare system" OR "health services" OR "health sector")

AND

("surgery" OR "surgical procedures" OR "surgical operations"

OR "operating room" OR "operating theatre"

OR "anesthesia" OR "anesthesiology"

OR "surgical waste"

OR "medical specialties" OR "specialist medicine" OR "medical departments"

OR “cardiology” OR “oncology” OR “orthopedics”

OR "intensive care" OR "ICU" OR "critical care"

OR "diagnostics" OR "laboratory tests" OR "laboratory diagnostics"

OR "laboratory analysis" OR "clinical diagnostics"

OR "radiology" OR "medical imaging" OR "diagnostic imaging"

OR "MRI" OR "magnetic resonance imaging"

OR "CT scan" OR "computed tomography"

OR "ultrasound" OR "sonography"

OR "pathology" OR "histopathology"

OR "pharmacology" OR "clinical pharmacology"

OR "pharmaceuticals"

OR "hospital departments" OR "hospital units")
