# Supplementary material for: The carbon footprint of surgical operations: 2023–2025 systematic review update
Source: PLoS One. 2026 May 18;21(5):e0349415. doi: 10.1371/journal.pone.0349415 (PMC13183196; doi:10.1371/journal.pone.0349415)
Supplement: S1 Table — (DOCX) [file pone.0349415.s003.docx]

**S1 Table. Quality Assessment.** 1°= primary, 2°= secondary, CI= confidence interval, GHG= greenhouse gas

| **Category** | **Scoring System** | **Kodumuri (2023)** | **Ramani (2023)** | **Rougerau (2023)** | **Zhang (2023)** | **Ahmed (2024)** | **Ang (2024)** | **Cannon (2024)** | **Eidmann(2024)** | **Filley (2024)** | **Grothaus (2024)** | **Karam (2024)** | **Kodumuri (2024)** | **Mousania (2024)** | **Nakarai (2024)** | **Parker (2024)** | **Shah (2024)** | **Spil (2024)** | **Ahmed (2025a)** | **Ahmed (2025b)** | **Camhi (2025)** | **Cohen (2025)** | **Drobetz (2025)** | **John (2025)** | **Mattei (2025)** | **Silva de Souza LC. (2025)** | **van Bree (2025)** |
| --- | --- | --- | --- | --- | --- | --- | --- | --- | --- | --- | --- | --- | --- | --- | --- | --- | --- | --- | --- | --- | --- | --- | --- | --- | --- | --- | --- |
| **1) Completeness** | a) To what extent are study inventory boundaries complete for a given functional unit? | 1 | 1 | 2 | 1 | 2 | 2 | 2 | 2 | 1 | 1 | 2 | 1 | 1 | 1 | 1 | 0 | 2 | 2 | 2 | 1 | 2 | 0 | 1 | 2 | 2 | 2 |
|  | Includes all reasonable factors (2) Includes limited/ ambiguous factors (1) Narrow focus (0) |  |  |  |  |  |  |  |  |  |  |  |  |  |  |  |  |  |  |  |  |  |  |  |  |  |  |
|  | b) Does the study account for all 3 scopes of GHG associated with the functional unit? | 0 | 0 | 2 | 1 | 2 | 1 | 2 | 2 | 0 | 1 | 2 | 1 | 1 | 2 | 0 | 0 | 2 | 2 | 2 | 2 | 2 | 0 | 1 | 2 | 2 | 2 |
|  | All 3 scopes measured (2) 2 scopes measured (1) scopes limited to 1 (0) |  |  |  |  |  |  |  |  |  |  |  |  |  |  |  |  |  |  |  |  |  |  |  |  |  |  |
| **2) Consistency** | a) To what extent is the study consistent with a recognised carbon footprinting guideline? | 1 | 1 | 2 | 1 | 1 | 2 | 2 | 2 | 2 | 0 | 2 | 2 | 1 | 2 | 0 | 0 | 0 | 2 | 1 | 1 | 2 | 0 | 2 | 2 | 2 | 2 |
|  | Stated and referenced (2) Stated, not referenced (1) No guideline stated (0) |  |  |  |  |  |  |  |  |  |  |  |  |  |  |  |  |  |  |  |  |  |  |  |  |  |  |
|  | b) For comparative studies, how consistently are methods applied? | 1 | 2 | 2 | 2 |  | 2 |  | 2 |  | 1 | 2 |  | 2 |  | 1 | 1 | 2 |  |  |  | 2 |  | 2 |  |  |  |
|  | Consistently applied throughout (2) Limited consistency (1) Poor consistency (0) |  |  |  |  |  |  |  |  |  |  |  |  |  |  |  |  |  |  |  |  |  |  |  |  |  |  |
| **3) Transparency** | a) Are the hypothesis(/es) and study objectives clearly stated? | 1 | 2 | 2 | 2 | 2 | 2 | 2 | 2 | 2 | 1 | 2 | 1 | 2 | 2 | 1 | 1 | 2 | 2 | 2 | 2 | 2 | 2 | 2 | 2 | 2 | 2 |
|  | Both clearly stated (2) Either hypothesis or study objectives stated (1) Neither stated (0) |  |  |  |  |  |  |  |  |  |  |  |  |  |  |  |  |  |  |  |  |  |  |  |  |  |  |
|  | b) To what extent are the GHGs included clearly stated? | 2 | 1 | 2 | 2 | 2 | 2 | 2 | 2 | 2 | 2 | 2 | 1 | 2 | 2 | 2 | 2 | 2 | 2 | 2 | 1 | 2 | 1 | 2 | 2 | 2 | 2 |
|  | Number of GHGs included clearly stated(2) Number of GHGs deducible (1) Number of GHGs not deducible (0) |  |  |  |  |  |  |  |  |  |  |  |  |  |  |  |  |  |  |  |  |  |  |  |  |  |  |
|  | c) To what extent are study assumptions and exclusions clearly stated? | 1 | 2 | 2 | 2 | 2 | 2 | 2 | 2 | 2 | 2 | 2 | 1 | 2 | 2 | 1 | 2 | 2 | 2 | 2 | 1 | 2 | 1 | 2 | 2 | 2 | 2 |
|  | Both assumptions and exclusions stated (2) Limited (1) Neither stated (0) |  |  |  |  |  |  |  |  |  |  |  |  |  |  |  |  |  |  |  |  |  |  |  |  |  |  |
|  | d) To what extent are the number of data points collected per process clearly stated? | 1 | 1 | 2 | 1 | 2 | 1 | 2 | 2 | 1 | 1 | 1 | 1 | 1 | 1 | 1 | 1 | 2 | 2 | 2 | 2 | 2 | 2 | 1 | 2 | 2 | 2 |
|  | Clear for all processes (2) Clear for limited processes (1) Not stated for any processes (0) |  |  |  |  |  |  |  |  |  |  |  |  |  |  |  |  |  |  |  |  |  |  |  |  |  |  |
|  | e) How transparent are reported GHG results? | 2 | 2 | 2 | 1 | 2 | 2 | 2 | 2 | 2 | 2 | 2 | 2 | 2 | 2 | 1 | 1 | 2 | 2 | 2 | 2 | 2 | 1 | 2 | 2 | 2 | 2 |
|  | Numerical data for all sub-processes (2) Limited numerical data for some sub-processes (1) Descriptive or graphical data only (0) |  |  |  |  |  |  |  |  |  |  |  |  |  |  |  |  |  |  |  |  |  |  |  |  |  |  |
| **4) Accuracy** | a) What is the specificity of the data sources to the study site? | 1 | 1 | 1 | 1 | 1 | 1 | 1 | 1 | 1 | 1 | 1 | 1 | 1 | 1 | 1 | 2 | 1 | 1 | 1 | 1 | 2 | 1 | 1 | 1 | 1 | 1 |
|  | 1° data only (2) Both 1° & 2° data (1) 2° data only (0) |  |  |  |  |  |  |  |  |  |  |  |  |  |  |  |  |  |  |  |  |  |  |  |  |  |  |
|  | b) Does the study determine parameter uncertainty? | 0 | 0 | 1 | 1 | 0 | 1 | 0 | 0 | 0 | 0 | 0 | 0 | 0 | 0 | 0 | 0 | 0 | 0 | 0 | 0 | 2 | 0 | 0 | 0 | 0 | 2 |
|  | Clear statistical plan with CI reported (2) CI reported, no clear plan (1) No CI or plan (0) |  |  |  |  |  |  |  |  |  |  |  |  |  |  |  |  |  |  |  |  |  |  |  |  |  |  |
|  | c) Does the study determine scenario uncertainty? | 0 | 0 | 0 | 0 | 1 | 0 | 0 | 0 | 2 | 0 | 0 | 0 | 1 | 0 | 0 | 0 | 0 | 1 | 0 | 0 | 2 | 0 | 2 | 0 | 0 | 2 |
|  | Yes, demonstrating minimal uncertainty (2) Yes, demonstrating large uncertainty (1) No (0) |  |  |  |  |  |  |  |  |  |  |  |  |  |  |  |  |  |  |  |  |  |  |  |  |  |  |
| **Total** | Percentage (scores out of 24 */22) (%) | 46% | 54% | 83% | 63% | 77% | 75% | 77% | 79% | 68% | 50% | 75% | 50% | 67% | 68% | 38% | 42% | 71% | 82% | 73% | 59% | 100% | 36% | 75% | 77% | 77% | 95% |
