# Supplementary material for: The carbon footprint of surgical operations: 2023–2025 systematic review update
Source: PLoS One. 2026 May 18;21(5):e0349415. doi: 10.1371/journal.pone.0349415 (PMC13183196; doi:10.1371/journal.pone.0349415)
Supplement: S2 Table — (DOCX) [file pone.0349415.s004.docx]

**S2 Table. Study inventory boundaries, data collected, and method used to calculate inventory results.**

| **Study** | **Process/item** | **Scope** | **Data source:data collected** | **Data type** | **Emission factor/global warming potential source** | **Impact assessment database** |
| --- | --- | --- | --- | --- | --- | --- |
| **Kodumuri et al. (2023)** | Surgical instruments | 3 | Direct measure: weight and material | Primary process data | Centre for Sustainable Healthcare | Nil |
|  | Consumables | 3 | Direct measure: weight and material | Primary process data | Centre for Sustainable Healthcare | Nil |
|  | Drapes | 3 | Direct measure: weight and material | Primary process data | Centre for Sustainable Healthcare | Nil |
|  | Sterilization |  | Centre for sustainable Healthcare data | Secondary process data | Centre for Sustainable Healthcare | Nil |
| **Ramani et al. (2023)** | Waste | 3 | Direct measure: Non reusable materials were weighted at the end of each procedure | Primary process data |  | Nil |
| **Rougerau et al. (2023)** | Transportation of staff | 3 | Hospital records and assumption: mode of transport, distance, assumed average commuting patterns | Primary process data | NHS England Carbon Emissions | Nil |
|  | Transportation of patient | 3 | Patient records: mode of transport, distance, estimated if missing data | Primary process data | NHS England Carbon Emissions | Nil |
|  | Electricity consumption: lighting, electrical equipment | 2 | Hospital facility reports: power specification, retrospective data on surgery time, estimated from literature if unavailable | Primary process data | ADEME dataset; NHS England Carbon Emissions | Nil |
|  | Heating, ventilation and air conditioning | 2 | Hospital data: average power specification and retrospective data on surgery time, estimated from national hospital benchmarks if missing | Primary process data | ADEME dataset | Nil |
|  | Water | 3 | Patient records: volume of wastewater per procedure, estimated from standard OR consumption rates | Primary process data | ADEME dataset | Nil |
|  | Consumables | 3 | Hospital procurement invoices: price paid per intervention | Primary monetary value data | NHS England Carbon Emissions, ecoinvent | Nil |
|  | Medical devices | 3 | Hospital procurement records: itemized breakdown of devices | Primary monetary value data | NHS England Carbon Emissions, ecoinvent | Nil |
|  | Laundry | 3 | Estimated per surgical procedure based on facility reports; Manufacturer data if unavailable | Secondary process data | ADEME dataset | Nil |
|  | Sterilization | 3 | Hospital data on sterilization cycles; Manufacturer energy & water consumption values used | Secondary process data | ADEME dataset | Nil |
|  | Pharmaceuticals | 3 | Hospital pharmacy logs: quantity per intervention; Estimated if no direct data available | Primary monetary value data | Literature | Nil |
|  | Anaesthesia | 1 | Hospital data: type of OR, duration of occupation; Gas volume per procedure recorded | Primary process data | Literature | Nil |
|  | Waste | 3 | Hospital waste audits: classified into WHR (household waste) and IRHW (infectious waste), then weighted | Primary process data | Not stated | Nil |
| **Zhang et al. (2023)** | Electricity consumption: lighting, electrical equipment | 2 | Hospital data and assumption: average power specification of each electronic equipment and surgery duration | Secondary process data | US Environmental Protection Agency | Nil |
|  | HVAC | 2 | Hospital data and assumption: averagepower utilization, averageoperating room dimension and retrospective data on surgery duration | Secondary process data | US Environmental Protection Agency | Nil |
|  | Central processing (sterilization) | 2 | Hospital data and assumption: averageenergy utiulization for one cycle, duration of one cycle and number of trays sterilized in one cycle | Secondary process data | US Environmental Protection Agency | Nil |
|  | Solid waste disposal | 3 | Hospital data: retrospective data on equipment catalogued during each surgery, weight from vendors and disposal modality | Primary process data | UK GHG Conversion factors | Nil |
| **Ahmed 2024** | Patient travel | 3 | Patient records: postcode-to-hospital distance; assumed petrol car; return journeys for all pre-surgery visits | Primary process data | GOV.UK 2023 Conversion Factors | Nil |
|  | Staff travel | 3 | Staff travel surveys: mode of transport and postcode-to-hospital distance; divided by patients per day | Primary process data | GOV.UK 2023 Conversion Factors; Ritchie H. (transport EFs) | Nil |
|  | Electricity, gas, oil | 2 | Annual hospital consumption from estates; minutes in clinical area x room proportion x per-minute consumption | Primary process data | GOV.UK 2023 Conversion Factors | Nil |
|  | Water | 3 | Annual hospital water consumption; operative-period usage doubled for resource-intensive setting | Secondary process data | GOV.UK 2023 Conversion Factors | Nil |
|  | Consumables and scrubs | 3 | Hospital records + literature; weight x emissions per kg | Secondary process data | Rizan et al. (2023); Shahmohammadi et al. (2018) | Nil |
|  | Medical devices (surgical equipment) | 3 | Hospital procurement data; direct observation of intraoperative equipment list | Secondary process data | Rizan et al. (2023) | Nil |
|  | Equipment transport | 3 | Supplier postcodes provided by estates; equipment weight x distance x freight truck factor | Secondary process data | Mathers J. (EDF green freight math) | Nil |
|  | CT angiogram | 2 | Hospital data; pre-surgery imaging | Secondary process data | Martin et al. (2018) | Nil |
|  | Anaesthesia (induction + maintenance + running equipment) | 1 | Anaesthesia records: induction agents (propofol/remifentanil); maintenance gases (N₂O + O₂ at 5 L/min); operative time | Primary process data | Narayanan et al. (2022); Association of Anaesthetists Impact Calculator (GWP100) | Nil |
|  | Sterilization of reusables | 3 | Central sterilisation unit: electricity (kWh) + water (m³) per tray x trays per procedure | Secondary process data | GOV.UK 2023 Conversion Factors | Nil |
|  | Laundry of gowns | 3 | Assumed one wash/procedure at 60 °C | Secondary process data | Shahmohammadi et al. (2018) | Nil |
|  | Waste management | 3 | Raw waste weighing (sharps, non-infectious offensive, DMR) per procedure | Primary process data | Rizan et al. (2021) | Nil |
|  | Surgical scrubbing | 3 | Minutes scrubbing x water per scrub x scrubs per procedure | Secondary process data | Cannings et al. (2022); GOV.UK 2023 | Nil |
| **Ang et al. (2024)** | Energy consumption | 2 | Hospital records: Operating room energy use estimated using hospital-wide energy data, with energy intensity assumptions based on NHS hospital benchmarks. | Secondary process data | DEFRA/BEIS | Nil |
|  | Consumables | 1 | Direct measurement: Tracked prospectively over a 3-week period using Material Flow Analysis (MFA) to record all disposable surgical consumables used per procedure. | Primary process data | WorldStainless, ICE v3 | Nil |
|  | Sterilization and Laundry | 2 | Hospital records + literature: Sterilization energy and water use obtained from hospital sterilization logs and supplemented with literature-based LCA data on reusable surgical instruments. | Secondary process data | Literature (Rizan et al. (2021)) | Nil |
|  | Waste management | 3 | Hospital records + literature: Waste composition analysed using hospital waste management reports, with emission factors for incineration and landfill taken from healthcare waste studies. | Secondary process data | Literature (Rizan et al. (2021)) | Nil |
|  | Transportation (patient/staff and surgical supply chain) | 2 | Logistics estimation: Supply chain distances for surgical consumables inferred from hospital procurement records, transport distances estimated using Google Maps and Pier2Pier tools. | Secondary process data | DEFRA/BEIS | Nil |
|  | Pharmaceuticals | 1 | Hospital records + government data: Pharmaceutical use estimated from patient records | Secondary process data | Centre for Sustainable Healthcare | Nil |
| **Cannon et al. (2024)** | Waste | 3 | Direct measurement: weight of infectious & mixed recycling waste | Primary process data | Literature, GOV.UK. Greenhouse gas reporting | Nil |
|  | Pharmaceuticals | 3 | Patient records: quantity used per procedure, duration of anaesthesia | Primary process data | Literature | Nil |
|  | Anaesthesia | 1 | Patient records: amount of sevoflurane used, average surgical duration | Primary process data | Literature | Nil |
|  | Sterilization | 3 | Hospital & literature data: transport distance, sterilization process emissions | Secondary process data | Literature, GOV.UK. Greenhouse gas reporting | Nil |
|  | Patient travel | 3 | Patient data: home postcode to hospital, distance travelled by petrol car | Secondary process data | GOV. UK GHG reporting | Nil |
|  | Staff travel | 3 | Survey: transport mode, distance, engine type and size | Secondary process data | GOV. UK GHG reporting | Nil |
|  | Consumables | 3 | Direct observation: quantity of surgical materials used | Primary process data | Literature | Nil |
|  | HVAC and lighting | 2 | Hospital data: energy consumption, theatre operating time | Secondary process data | GOV. UK GHG reporting | Nil |
|  | Electronic equipment |  | Hospital data: power consumption from manufacturer specification documents | Secondary process data | GOV. UK GHG reporting | Nil |
| **Eidmann et al. (2024)** | Anaesthesia | 1 | Anaesthesia protocol (minimal-flow: 4 L/min x 10 min, then 0.5 L/min); Anaesthetic Impact Calculator (RCoA) | Secondary process data | Royal College of Anaesthetists - Anaesthetic Impact Calculator; IPCC GWP100 factors | Not specified beyond GHG Protocol |
|  | HVAC | 2 | Manufacturer specs (Robatherm, Smardt OPK); German Weather Service TRY dataset; hospital CHP system | Secondary process data | German Federal Office CO₂ factors | GHG Protocol |
|  | Electricity consumption (OR devices) | 2 | Hospital technical services: equipment list & consumption rates (per min × duration) | Secondary process data | Local electricity company (WVV Würzburg 2022 mix) | GHG Protocol |
|  | Sterilization | 2 | Hospital sterilization unit logs; device specifications (Miele PG 8528; MMM Selectomat) | Secondary process data | Manufacturer data + hospital energy factors (gas/electricity) | GHG Protocol |
|  | Waste disposal | 3 | Hospital waste records; incineration at municipal WtE plant; glass recycling | Primary process data | Incineration facto (WVV Umwelt 2022) | GHG Protocol |
|  | Consumables and Disposables | 3 | Waste composition back-calculated into material categories (90% mixed plastics, 10% cardboard; sharps = glass, steel, plastic) | Secondary process data | German Federal Office for Economics: plastics 3.7; cardboard 0.62; glass 1; steel 5.18 (kg CO₂/kg) | GHG Protocol |
|  | Laundry | 3 | Outsourced laundry provider ESG report (Ullmer-Gruppe 2022) | Secondary process data | Provider data | GHG Protocol |
| **Filley et al. (2024)** | Consumables | 3 | Hospital data; quantity, type | Secondary process data | Ecoinvent | ReCiPe |
|  | Reusables | 3 | Direct measure; quantity, weight, lifetime | Primary process data | Ecoinvent | ReCiPe |
|  | Sterilization | 3 | Hospital data; manufacturer instructions-for-use, quantity of energy and water for each cycle | Secondary process data | Ecoinvent | ReCiPe |
| **Grothaus et al. (2024)** | Disposables | 3 | Hospital data: cost and quantity of disposable instruments per procedure | Primary financial data | Practice Greenhealth | Nil |
|  | HVAC and lighting | 2 | Hospital data: annual energy consumption for OR vs PR | Secondary process data | US EPA | Nil |
|  | Waste | 3 | Hospital data and literature: weight of reusable items and disposable waste generation per procedure | Secondary process data | US EPA | Nil |
|  | Sterilization | 3 | Hospital data and literature; weights of reusable items and average consumption of steam sterilisation per kg | Secondary process data | US EPA | Nil |
| **Karam et al. (2024)** | Electricity consumption: lighting, electrical equipment | 2 | Hospital facility data; manufacturer power specifications | Primary process data | Not stated | ReCiPe 2016 |
|  | HVAC | 2 | Facility averages, retrospective OR energy time per procedure | Primary process data | Not stated | ReCiPe 2016 |
|  | Water consumption |  | Estimated from scrubbing, draping, and sterilization reports | Secondary process data | Not stated | ReCiPe 2016 |
|  | Consumables and Reusables | 3 | Hospital procurement invoices and hospital inventory | Primary financial data | Not stated | ReCiPe 2016 |
|  | Sterilization and Laundry | 3 | Sterilization unit energy and water data (manufacturer values) | Secondary process data | Not stated | ReCiPe 2016 |
|  | Pharmaceuticals | 3 | Hospital records | Primary process data | Not stated | ReCiPe 2016 |
|  | Waste management | 3 | Hospital waste audit data and waste management contractor data | Primary process data | Not stated | ReCiPe 2016 |
| **Kodumuri et al. (2024)** | Consumables | 3 | Hospital data and direct measure: quantity, weight, materials | Primary process data | Centre for Sustainable Healthcare | Nil |
|  | Reusables | 3 | Hospital data and direct measure: quantity, weight, materials | Primary process data | Centre for Sustainable Healthcare | Nil |
|  | HVAC and lighting | 2 | Hospital data: volume, duration of surgery, power specification | Secondary process data | Centre for Sustainable Healthcare | Nil |
|  | Water | 2 | Direct measure: total water flow measured with a full tap flow system | Primary process data | Centre for Sustainable Healthcare | Nil |
|  | Sterilization | 3 | Estimation and hospital data; number of cycle, power consumption | Secondary process data | Centre for Sustainable Healthcare | Nil |
|  | Waste | 3 | Direct measure: type and weight | Primary process data | Centre for Sustainable Healthcare | Nil |
| **Mousania et al. (2024)** | Electricity | 2 | Hospital records: power ratings and duration of use for surgical devices measured per procedure. | Secondary process data | Ecoinvent, USLCI | IPCC 2021 GWP-100a, Available WAter REmaining (AWARE) |
|  | Central processing (sterilization and decontamination) | 2 | Hospital sterilization logs: energy and water consumption per decontamination cycle, chemical composition of sterilization agents (propylene glycol, borax, subtilisin), manufacturer manuals information | Secondary process data | Ecoinvent, USLCI | IPCC 2021 GWP-100a, Available WAter REmaining (AWARE) |
|  | Solid waste (landfill/incineration) | 3 | Hospital waste reports: classification and weight of single-use item waste streams, treatment processes (landfill/incineration) based on waste disposal records. | Primary process data | Ecoinvent, USLCI | IPCC 2021 GWP-100a, Available WAter REmaining (AWARE) |
|  | Transportation of materials (pre- and post-consumer) | 2 | Transport logistics data: supplier-to-hospital distances obtained from Google Earth; fuel consumption and emissions factors from transportation studies. | Secondary process data | Ecoinvent, USLCI | IPCC 2021 GWP-100a, Available WAter REmaining (AWARE) |
|  | Material production | 1 | Hospital records; quantities, material and lifecycle information | Primary process data | Ecoinvent, USLCI | IPCC 2021 GWP-100a, Available WAter REmaining (AWARE) |
| **Nakarai et al. (2024)** | Disposables | 3 | Medical records and direct measure: weights and packaging | Secondary process data | Ecoinvent | Nil |
|  | Reusables | 3 | Medical records and direct measure: weights, packaging, number of uses | Secondary process data | Ecoinvent | Nil |
|  | Transport of instruments | 3 | Assumption: type of transport and distance | Secondary process data | Ecoinvent | Nil |
|  | HVAC, lighting and electronic equipment | 2 | Hospital data and literature: average energy consumption and duration of surgery | Secondary process data | Ecoinvent | Nil |
|  | Anaesthesia | 1 | Medical records: specific equation to calculate quantity | Secondary process data | Literature | Nil |
|  | Sterilization | 3 | Literature and hospital data: electricity consumption per kg | Secondary process data | Ecoinvent | Nil |
|  | Waste | 3 | Literature and hospital data: type of waste and disposal process | Secondary process data | Literature | Nil |
| **Parker et al. (2024)** | Consumables and Disposables | 3 | Direct measure: weight linked to waste | Primary process data | MacNeill et al. (2017), Rubio-Domingo G. et al. (2023) | Nil |
|  | Waste | 3 | Direct measure: weight | Primary process data | MacNeill et al. (2017), Rubio-Domingo G. et al. (2023) | Nil |
|  | Sterilization | 3 | Hospital data: instrument tray size, washer & autoclave cycle duration and energy consumed per cycle | Secondary process data | US Environmental Protection Agency | Nil |
|  | Laundry | 3 | Literature: average consumption of hospital laundry | Secondary process data | US Environmental Protection Agency based on industrial laundry emissions factors | Nil |
| **Shah et al. (2024)** | Waste | 3 | Waste audit and hospital policy: weight, type of waste and mode of disposal, processing | Primary process data | Emission factor obtained from waste contractor | NIl |
| **Spil et al. (2024)** | Electricity consumption | 2 | Observational audit (Manchester & Leiden maternity units); expert review for instrument trays and devices. | Primary process data | Idemat 2023 database (material & production) | OpenLCA v1.11.0; *Eco-costs 2023 (Sustainability Impact Metrics)* |
|  | HVAC | 2 | Hospital energy certificates (Saint Mary’s Hospital DEC; CIBSE energy benchmarks). | Primary process data | DEFRA 2023 energy emission factors | OpenLCA v1.11.0; *Eco-costs 2023 (Sustainability Impact Metrics)* |
|  | Water | 3 | Included via sterilization and laundry processes. | Secondary process data | ADEME / HealthcareLCA (assumed from reused studies) | OpenLCA v1.11.0; *Eco-costs 2023 (Sustainability Impact Metrics)* |
|  | Consumables | 3 | Waste audits + material weighing of consumables and PPE (UK & NL); Estimated transport to hospital. | Primary and Secondary process data | Idemat 2023; DEFRA; Inventory of Carbon and Energy (ICE). | OpenLCA v1.11.0; *Eco-costs 2023 (Sustainability Impact Metrics)* |
|  | Reusables | 3 | Weighed instruments; assumed 50 reuses per tray (tested at 100-300 in sensitivity analysis). | Primary process data | Idemat 2023; HealthcareLCA | OpenLCA v1.11.0; *Eco-costs 2023 (Sustainability Impact Metrics)* |
|  | Reprocessing | 3 | Hospital on-site sterilization data; supplemented by HealthcareLCA. | Secondary process data | Rizan et al. (2022); Idemat 2023. | OpenLCA v1.11.0; *Eco-costs 2023 (Sustainability Impact Metrics)* |
|  | Laundry | 3 | Emission factors from Rizan et al. (2021). | Secondary process data | Rizan et al. (2021) (UK dataset). | OpenLCA v1.11.0; *Eco-costs 2023 (Sustainability Impact Metrics)* |
|  | Anaesthesia | 3 | Analgesia/anaesthesia drugs (nitrous oxide, remifentanil, morphine, epidural) from literature; Hospital data + national frequency datasets (UK Care Quality Commission; Perined NL). | Secondary process data | Pearson et al. (2022); Parvatker et al. (2019) | OpenLCA v1.11.0; *Eco-costs 2023 (Sustainability Impact Metrics)* |
|  | Waste management - disposal | 3 | Offensive waste → low-temp incineration w/ energy recovery; sharps & drugs → high-temp incineration. | Primary and Secondary process data | Rizan et al. (2021); Idemat 2023 | OpenLCA v1.11.0; *Eco-costs 2023 (Sustainability Impact Metrics)* |
| **Ahmed 2025a** | Patient travel | 3 | Patient records: postcode-to-hospital distance x 13 visits across pathway; return journeys; petrol car assumption; international patients excluded | Primary process data | GOV.UK 2023 Conversion Factors | Nil |
|  | Staff travel | 3 | Staff travel surveys: mode + distance; divided by patients per day | Primary process data | GOV.UK 2023 Conversion Factors; Ritchie H. | Nil |
|  | Electricity, gas, oil | 2 | Annual hospital consumption; minutes x room proportion x per-minute factor | Primary process data | GOV.UK 2023 Conversion Factors | Nil |
|  | Water | 3 | Hospital consumption; operative-period doubled | Secondary process data | GOV.UK 2023 Conversion Factors | Nil |
|  | Consumables and scrubs | 3 | Hospital records + literature; weight x emissions per kg | Secondary process data | Rizan et al. (2023); Shahmohammadi et al. (2018) | Nil |
|  | Medical devices (surgical equipment) | 3 | Procurement + intraoperative direct observation (stage 1 & stage 2) | Secondary process data | Rizan et al. (2023) | Nil |
|  | Equipment transport | 3 | Supplier postcodes; weight x distance x freight factor | Secondary process data | Mathers J. (EDF green freight math) | Nil |
|  | Anaesthesia (induction + maintenance + running equipment) | 1 | Anaesthesia records: stage 1 propofol + remifentanil + sevoflurane + N₂O; stage 2 propofol + remifentanil + sevoflurane only | Primary process data | Narayanan et al. (2022); Association of Anaesthetists Impact Calculator (GWP100) | Nil |
|  | Sterilization of reusables | 3 | Central sterilisation unit: electricity + water per tray | Secondary process data | GOV.UK 2023 Conversion Factors | Nil |
|  | Laundry of gowns | 3 | One wash per procedure at 60 °C | Secondary process data | Shahmohammadi et al. (2018) | Nil |
|  | Waste management (operative) | 3 | Raw weighing of stage 2 waste; stage 1 scaled by suture needles (sharps), swabs (non-infectious offensive), staff (DMR) | Primary + secondary process data | Rizan et al. (2021) | Nil |
|  | Surgical scrubbing | 3 | Minutes x water per scrub x scrubs per procedure | Secondary process data | Cannings et al. (2022); GOV.UK 2023 | Nil |
|  | Inpatient stay (2 admissions) | 1, 2, 3 | Top-down approach: NHS England per-inpatient-bed-day framework (89.5 kgCO₂e/day); high-intensity postoperative patient assumed | Secondary process data | NHS England (2015) Sustainable Care Pathways Guidance | Nil |
|  | Dressing clinics (4 total) | 3 | Patient and staff travel to nurse-led outpatient clinics; building electricity/gas/oil | Primary + secondary process data | GOV.UK 2023 Conversion Factors | Nil |
| **Ahmed 2025b** | Patient travel | 3 | Patient records: postcode-to-hospital distance; assumed petrol car | Primary process data | GOV.UK 2023 Conversion Factors | Nil |
|  | Staff travel | 3 | Staff travel surveys: mode + distance; divided by patients per day | Primary process data | GOV.UK 2023 Conversion Factors; Ritchie H. | Nil |
|  | Electricity, gas, oil | 2 | Annual hospital consumption from estates; per-minute allocation | Primary process data | GOV.UK 2023 Conversion Factors | Nil |
|  | Water | 3 | Hospital consumption; operative-period doubled | Secondary process data | GOV.UK 2023 Conversion Factors | Nil |
|  | Consumables and scrubs | 3 | Hospital records + literature; weight x emissions per kg | Secondary process data | Rizan et al. (2023); Shahmohammadi et al. (2018) | Nil |
|  | Medical devices (surgical equipment) | 3 | Procurement + direct observation of intraoperative list; 1 supplier provided individual LCA | Primary + secondary process data | Rizan et al. (2023); supplier LCA | Nil |
|  | Equipment transport | 3 | Supplier postcodes; weight x distance x freight factor | Secondary process data | Mathers J. (EDF green freight math) | Nil |
|  | Anaesthesia (induction + running equipment) | 1 | Anaesthesia records: TIVA (propofol + remifentanil); no volatile maintenance gases; running anaesthetic equipment | Primary process data | Narayanan et al. (2022) | Nil |
|  | Sterilization of reusables | 3 | Central sterilisation unit: electricity + water per tray x trays per procedure | Secondary process data | GOV.UK 2023 Conversion Factors | Nil |
|  | Laundry of gowns | 3 | One wash per procedure at 60 °C | Secondary process data | Shahmohammadi et al. (2018) | Nil |
|  | Waste management | 3 | Raw waste weighing (sharps, non-infectious offensive, DMR) | Primary process data | Rizan et al. (2021) | Nil |
|  | Surgical scrubbing | 3 | Minutes x water per scrub x scrubs per procedure | Secondary process data | Cannings et al. (2022); GOV.UK 2023 | Nil |
| **Camhi et al. (2025)** | Surgical equipment | 3 | Direct observation of surgical procedures; weighing of each instrument and material (with/without packaging); categorization by material (plastic, paper, metal); manufacturer data for reusable items | Primary process data | ADEME CARBONE database v22.0; French energy mix 2021 | Nil |
|  | Anaesthesia drugs | 1 | Hospital anaesthesia records: quantity and type of drugs used per procedure | Primary process data | ADEME CARBONE database v22.0 | Nil |
|  | Energy consumption | 2 | Manufacturer data (power specifications), hospital sterilization logs for reusable instruments, Climat Mundi calculations | Secondary process data | ADEME CARBONE database v22.0 | Nil |
|  | Sterilization and decontamination | 3 | Hospital sterilization unit data; manufacturer literature when missing | Secondary process data | ADEME CARBONE database v22.0 | Nil |
|  | Transportation of medical devices | 3 | Inventory of production sites; assumption of sea freight (container) and road freight (truck) | Secondary process data | ADEME CARBONE database v22.0 | Nil |
|  | Waste (incineration/disposal) | 3 | Direct measurement of device weights after procedure; disposal modality considered | Primary process data | ADEME CARBONE database v22.0 | Nil |
| **Cohen et al. (2025)** | Waste | 3 | 40 prospective waste inventories; >13,500 items sorted, weighed, classified; hospital documentation & manufacturers | Primary process data | Ecoinvent v3.10 | ReCiPe 2016 |
|  | HVAC and medical equipment | 2 | Hospital utility records (electricity, gas, steam, cooling); hospital engineer HVAC model; plug load smart meters | Primary and Secondary process data | Dutch cogeneration mix; local electricity grid; Ecoinvent v3.10; Van den Berg (2023) | ReCiPe 2016 |
|  | Hospital stay | 2 | Allocation of energy/water per floor area & patient time; hospital facility management records | Secondary process data | Dutch energy provider mix; cogeneration facility efficiency (internal docs) | ReCiPe 2016 |
|  | Staff and patient travel | 3 | Hospital sustainability report; patient records (postcodes, modes, distance); staff allocation per FTE | Secondary process data | DEFRA 2022 emission factors (modal split car, train, bike, bus) | ReCiPe 2016 |
|  | Sterilization and washing | 3 | Literature data; washer/autoclave parameters; detergent composition | Secondary process data | Ecoinvent v3.10 | ReCiPe 2016 |
|  | Textiles / Laundry (gowns, linen, headcovers) | 3 | Waste inventory weights; literature data; allocation per use | Secondary process data | Ecoinvent v3.10; literature (ADEME, Rizan 2021) | ReCiPe 2016 |
|  | Consumables | 3 | Prospective waste inventory classification; manufacturer info; assumptions by largest material weight (plastic type) | Primary process data | Ecoinvent v3.10 unit processes | ReCiPe 2016 |
|  | Pharmaceuticals | 1 | Patient records; median dosages; literature cradle-to-gate APIs | Primary + secondary | Literature + ecoinvent (if available); excluded APIs where no data | ReCiPe 2016 |
|  | Waste | 3 | Hospital waste managers; MSW incineration; RMW treatment; recycling routes (cotton, steel, plastics) | Primary + secondary | Ecoinvent v3.10 + PEF Circular Footprint Formula allocation | ReCiPe 2016 |
| **Drobetz et al. (2025)** | Waste measurement: landfill, recycling, biohazard | 3 | Prospective hospital data collection; waste weighed to ±10 g | Primary process data | Australian National Greenhouse Accounts Factors | None beyond EF conversion |
| **John et al. (2025)** | Patient and staff travel | 3 | Hospital data: averageround trip distance, modes of transport | Secondary process data | DEFRA, BEIS | Nil |
|  | Gas and Electricity | 2 | Hospital data and assumption: annual use divide per hospital area and patients receiving the service | Secondary process data | DEFRA, BEIS | Nil |
|  | Water | 3 | Hospital data and assumption: data for the main hospital divided to floor area and patient volume | Secondary process data | DEFRA, BEIS | Nil |
|  | Sterilization | 3 | Hospital data, literature and assumptions; type of decontamination, power specification by manufacturer, boiler characteristics by literature, estimation on duration and water consumption of each cycle/load | Secondary process data | DEFRA, BEIS | Nil |
|  | Laundry | 3 | Hospital data; Consumption of gas, electricity, water and fuel oil data, quantity of detergent, mass of laundering different linen | Secondary process data | DEFRA, BEIS | Nil |
|  | Consumables and medical equipment | 3 | Direct measure, estimation and manufacturer data: weight, composition and LCA by manufacturer | Secondary process data | DEFRA, BEIS | Nil |
|  | Anaesthesia | 1 | Direct measure: volume of gas used for 5 patients | Primary process data | DEFRA, BEIS | Nil |
|  | Waste |  | Direct measure and observation: mass and type of product dispose of | Primary process data | DEFRA, BEIS | Nil |
| **Mattei et al. (2025)** | Patient transportation | 3 | Google Maps distance home-hospital × 5 trips; all patients assumed petrol car | Secondary process data | Carebone tool (AP-HP, Paris); French emission factors | Carebone (embedded LCA db) |
|  | Consultations | 3 | Included in 5 round trips (pre-op + surgery + 3 post-op) | Secondary process data | Carebone | Carebone |
|  | Consumables | 3 | OR database; list of disposables per case | Primary process data | Carebone | Carebone |
|  | Medications | 3 | OR pharmacy logs; dose per patient | Primary process data | Carebone | Carebone |
|  | Anaesthesia | 1 | Anaesthesia records; mean 38 g per case | Primary process data | IPCC AR5 GWP100 values (sevoflurane GWP100 = 130) | Carebone |
|  | Medical devices (single-use + reusables) | 3 | OR procurement data; detailed per procedure | Primary process data | Carebone | Carebone |
|  | Electricity consumption | 2 | Hospital OR logs | Secondary process data | French electricity mix (70% nuclear, INSEE data) | Carebone |
|  | Heating | 2 | Hospital OR logs | Secondary process data | French heating energy factors | Carebone |
|  | Sterilization | 3 | Hospital sterilization department data, adjusted to number of uses | Secondary process data | Carebone | Carebone |
| **Silva de Souza Lima Cano et al. (2025)** | Electricity consumption | 2 | Hospital equipment inventory; manufacturer specifications; wattage; duration of ACLR | Secondary process data | US EIA 2022 Pennsylvania electricity mix; USLCI and Ecoinvent 3.8 | TRACI 2.1 |
|  | HVAC | 2 | Campion bin model updated with Pittsburgh 2022 weather; OR occupancy and volume data | Secondary process data | US EPA, USLCI | TRACI 2.1 |
|  | Reusable surgical instruments | 3 | Hospital records; 3 trays sterilized per ACLR; washer/autoclave logs; detergent/enzymes | Secondary process data | Ecoinvent 3.8 | TRACI 2.1 |
|  | Reusable cotton gowns (laundry) | 3 | 9 gowns (patient+staff); Campion laundry model with 350 km roundtrip to facility; detergent composition | Secondary process data | Literature; Ecoinvent 3.8 | TRACI 2.1 |
|  | Disposable consumables | 3 | Material flow analysis (MFA) with direct weighing and manufacturer info; SankeyMATIC mapping | Primary process data | Ecoinvent 3.8 unit processes | TRACI 2.1 |
|  | Material production | 1 | Manufacturer specifications and assumptions (single material per product); exclusions for hydrogel, wood swabs | Secondary process data | Ecoinvent 3.8 | TRACI 2.1 |
|  | Waste disposal | 3 | Hospital waste logs; landfill transport assumptions | Primary process data | US EPA factors | TRACI 2.1 |
| **van Bree et al. (2025)** | Electricity consumption | 2 | Hospital records; manufacturer specifications; average CABG duration | Secondary process data | Dutch national electricity mix (2019-2021); Ecoinvent 3.9.1 | ReCiPe 2016 Midpoint |
|  | HVAC | 2 | Modelled with number of air changes/h; OR occupancy and room size; duration per CABG | Secondary process data | Dutch benchmarks for hospital HVAC; Ecoinvent 3.9.1 | ReCiPe 2016 Midpoint |
|  | Surgical instruments | 3 | Hospital procurement data; sterilization logs; washer/autoclave specifications | Primary process data | Ecoinvent 3.9.1 | ReCiPe 2016 Midpoint |
|  | Laundry | 3 | Hospital laundry facility data; detergent composition; estimated number of items per surgery | Secondary process data | Literature (Rizan 2021; ADEME); Ecoinvent 3.9.1 | ReCiPe 2016 Midpoint |
|  | Disposables & consumables | 3 | Procurement invoices; direct observation of surgeries; manufacturer composition data | Primary process data | Ecoinvent 3.9.1 | ReCiPe 2016 Midpoint |
|  | Pharmaceuticals | 3 | Hospital pharmacy logs; dosages per CABG; packaging mass | Primary process data | Ecoinvent 3.9.1 | ReCiPe 2016 Midpoint |
|  | Anaesthetic gases | 1 | Anaesthesia records: gas volume per case; average flow rates | Primary process data | IPCC AR5 GWP100 values | ReCiPe 2016 Midpoint |
|  | Staff commuting | 3 | Surveys and hospital HR data; average commuting distance; mode split | Secondary process data | DEFRA 2022 emission factors | ReCiPe 2016 Midpoint |
|  | Patient travel | 3 | Patient records: postcode to hospital distance; mode of transport assumptions | Secondary process data | DEFRA 2022 emission factors | ReCiPe 2016 Midpoint |
|  | Waste treatment & disposal | 3 | Hospital waste audits; mass and type of infectious vs household waste; landfill vs incineration | Primary process data | Ecoinvent 3.9.1; Dutch waste contractor factors | ReCiPe 2016 Midpoint |
|  | Water consumption | 3 | Facility water logs; average CABG water use per procedure (sterilization + cleaning) | Secondary process data | Ecoinvent 3.9.1; Dutch water treatment data | ReCiPe 2016 Midpoint |
