# Supplementary material for: The carbon footprint of surgical operations: 2023–2025 systematic review update
Source: PLoS One. 2026 May 18;21(5):e0349415. doi: 10.1371/journal.pone.0349415 (PMC13183196; doi:10.1371/journal.pone.0349415)
Supplement: S3 Table — (DOCX) [file pone.0349415.s005.docx]

**S3 Table. Stated exclusions, assumptions and other limitations.**

| **Study** | **Stated exclusions of boundary; functional unit, processes** | **Stated assumptions in data collection** | **Other limitations** |
| --- | --- | --- | --- |
| **Kodumuri (2023)** | Electricity consumption; HVAC; waste | Carbon emission of sterilization of generic hand tray is calculated via Centre for Sustainable Healthcare data | Stated: Declare to calculate only Scope 1 emissions, but the emission factors related to materials and instruments, are referred to Scope 3 emissions. No one of the factors considered is responsible of Scope 1 emissions.  Not Stated: No parameter uncertainty or scenario uncertainty. |
| **Ramani (2023)** | Scope 1 and 2 emissions were excluded, meaning: hospital-wide electricity and HVAC energy use were not measured. operating room lighting, ventilation, and infrastructure emissions were omitted. Scope 3 was only partially considered, as manufacturing, transportation, and disposal emissions of medical devices were not included, pharmaceutical emissions (e.g., anaesthesia) were omitted. Sterilization energy for reusable instruments was not accounted for. No consideration for water usage or hospital-wide waste management impacts. | Only non-reusable waste was weighed. No differentiation between landfill, incineration, or recycling. EPA carbon emission factors were applied to all surgical waste without process-specific adjustments. | Stated: The study only considers non-reusable waste emissions and excludes full LCA factors such as hospital energy, transportation, and sterilization processes. Data is from one U.S. hospital, limiting generalizability. Only three VH cases were included, reducing statistical reliability.  Not stated: The study does not follow a full cradle-to-grave LCA methodology and only evaluates surgical waste CO₂ emissions. This study omits anaesthesia-related emissions, which are major contributors to surgical CF. This study omits anaesthesia-related emissions, which are major contributors to surgical CF. The study assumes all waste contributes equally to CO₂ emissions, though incineration, landfill, and recycling have different carbon impacts. The study assumes all waste contributes equally to CO₂ emissions, though incineration, landfill, and recycling have different carbon impacts. |
| **Rougerau (2023)** | Heating emissions at night excluded( hospital heating and lighting are reduced at night), the manufacture, maintenance, or potential repairs of the Neptune® surgical waste management system​ | Energy use estimation d per operating room and per procedure, assuming a constant rate of energy use per hour; Surgical waste was divided into household refuse (WHR) and infectious risk healthcare waste (IRHW), assuming consistent disposal practices. Standardized patient and staff transport emissions; pre-existing emission factors (e.g., 0.315 kgCO₂eq/euro for implants, 0.5403 kgCO₂eq/euro for pharmaceuticals) without specific hospital-based assessments. | Stated: The study was conducted in one hospital, limiting its applicability to other healthcare settings. The study was conducted in one hospital, limiting its applicability to other healthcare settings. Limited impact of sustainability interventions: While locoregional anaesthesia, outpatient surgery, and water filtration reduced emissions by 12.2%, the study acknowledges that the largest emissions sources (single-use implants and instruments) remain unchanged.  Not stated: No breakdown of surgical consumables: while waste is categorized as disposable vs. reusable, the study does not analyse which specific items contribute most to emissions. Lack of alternative implant assessment: The study does not evaluate whether alternative implants (e.g., biodegradable, metal vs. polymer) could reduce emissions. No assessment of hospital-wide sustainability initiatives: Other potential measures like recycling, solar energy integration, or surgical tray optimization were not considered. |
| **Zhang (2023)** | Transportation of patients to and from the hospital; transportation of staff to and from the hospital; laundering of scrubs; production of disposable supplies | Energy usage assumed to be the same of the facility and equipment, as well as central processing capabilities; Only retrospective data; | Stated: Results are based on estimations rather than direct operational measurements. Energy allocation methods were assumed rather than precisely tracked. Use UK GHG emission factors for disposal of solid waste and not US emission factors.  Not stated: No parameter uncertainty or scenario uncertainty |
| Ahmed (2024) | Postoperative period (inpatient stay + follow-up) due to highly variable nature; long-term complications; revisional procedures; manufacturing/supply-chain emissions of hospital infrastructure; immediate postoperative recovery included but limited to building energy only. Functional unit: one patient undergoing unilateral DIEP flap surgery from first plastic surgery encounter through immediate postoperative recovery. | Patient/staff travel assumed one-way distance using FreeMapTools with return journeys; mode of transport based on staff survey; patient transport assumed petrol car; equipment transport modeled via supplier postcode x weight x freight truck emissions (EDF green freight math); anaesthetic emissions calculated from drug doses x average patient weight x operative time (Narayanan et al. 2022); CT angiogram emissions from Martin et al. 2018; sterilization energy/water per tray from Central Sterilisation Unit x trays per procedure; one laundry load per procedure at 60 °C; waste emission factors from Rizan et al. 2021. | Stated: single-centre retrospective study (n=42) limiting generalizability; reasonable estimates used from existing literature as benchmarks when primary data unavailable; product-level LCA data requested from external suppliers but only 1 anaesthetic equipment supplier responded; variability between surgeons in equipment preferences and operative time (mean range 270 min); differences in unipedicled vs bipedicled DIEPs not deeply explored; postoperative period excluded due to variability. Not stated: no parameter uncertainty or confidence intervals reported; no formal sensitivity analysis beyond "what-if" TIVA scenario (estimated 38% reduction); no assessment of long-term implant/revisional carbon impact; cradle-to-gate boundary for some equipment (disposal emissions beyond supplier limited). |
| **Ang (2024)** | Staff and patient transport, manufacture of reusable surgical instruments and machinery used, delivery of reusable instruments and machinery | Operating theatre energy use was modelled at 3-6 times the hospital’s average floor-space consumption. All surgical materials were assumed to be shipped via sea freight. Carbon emissions from incineration, landfill, and recycling were modelled using published UK hospital data. Steam sterilization emissions were based on prior UK hospital studies rather than direct measurement. | Stated: The analysis was restricted to intraoperative emissions, excluding preoperative and postoperative activities such as outpatient visits, diagnostic testing, and patient recovery. Patient care outside of the operating theatre (e.g., follow-up visits, wound management) was not included in the carbon footprint calculations. The lack of individual energy meters for the operating theatre meant that energy usage had to be estimated rather than directly measured. The ICE (Inventory of Carbon and Energy) database used for emission factors follows a "cradle-to-gate" model, meaning that emissions beyond the manufacturing and transportation stages (e.g., disposal or recycling) were not included. The study was conducted at a single plastic surgery centre, and its findings may not be directly transferable to other hospitals, surgical units, or geographical locations.  Not stated: While sterilization emissions are included, the study does not account for the entire life cycle of reusable surgical instruments, including repair, refurbishment, and eventual disposal. Hospital-specific protocols for waste management and sterilization may lead to variations in carbon footprint not captured in this single-centre study. |
| **Cannon (2024)** | Production and disposal of the infrastructure (machines, means of transportation), the need for repeat operations, preoperative assessment and post-operative care, staff training or any supportive services, attributable processes reasonably thought to contribute less than 1% of GHG; pre-operative and post-operative care | Waste weight based on weighing bags before and after surgery, assuming accurate disposal practices, pharmaceutical emissions calculations derived from literature on chemical synthesis and processing, with proxy data used for diclofenac and ketorolac, surgical instrument decontamination using published sterilization emission factors, assuming standard sterilization cycles, transport emissions calculated from home-hospital distance using Google Maps, assuming all patients travelled by petrol cars/taxis, staff travel emission based on a survey sample (n=13), proportionally attributed to each tonsillectomy based on daily operating time. | Stated: reliance on secondary data sources, individual transport modes and distances vary significantly but were estimated using averages, only HVAC and lighting were directly measured, plug load estimates were based on manufacturer data, emissions from production vs. disposal of materials were not separated, drug wastage not accounted for.  Not stated: environmental trade-offs not considered, for example, the impact of switching from sevoflurane to propofol is mentioned but not explored in-depth, limited applicability beyond ENT surgery |
| **Eidmann (2024)** | Excluded: patient & staff travel; pharmaceuticals (except sevoflurane); implants production; idle time in surgical area; hospital infrastructure. | - Minimal-flow sevoflurane anaesthesia protocol assumed standard across all cases. - Waste composition assumed: 90% mixed plastics, 10% cardboard; sharps = equal parts glass, plastic, steel. - Heating required 87% of year, AC 13% based on TRY dataset. - Supply chain emissions estimated from material composition of waste. | Stated: Small case numbers (n=5 per procedure); calculations site-specific (Germany, Würzburg electricity mix, weather, infrastructure); implant production not traceable; pharmaceuticals largely excluded.  Not stated: No parameter or scenario uncertainty; no sensitivity analysis on waste composition or energy mix; no evaluation of variability across hospitals. |
| **Filley (2024)** | Anaesthetic medications and electronic equipment, building operation (e.g., HVAC, lighting), and labour, wastewater treatment; no variations of the functional unit across different hospital; manufacturing and transportation of certain medical equipment are not detailed if already included in the LCA database | Optimal efficiency for decontamination is assumed, reusable surgical instruments are assumed to have a lifespan of 4000 uses with their environmental impact amortized over this period, assumption on single-use devic3d transportation via cargo ship | Stated: The study is limited to a single hospital and may not be generalizable to other institutions. Differences in clinical protocols across hospitals or countries are not considered. Water and energy consumption data are based on average values and may not reflect real-world variability in all settings.  Not stated: The comparison between single-use and reusable instruments does not consider the long-term economic cost of both alternatives. The impact on patient safety from reducing sterile materials is not analysed. |
| **Grothaus (2024)** | Patient and staff travel; anaesthesia, electronic equipment, water; preoperative and postoperative procedures | Disposable item weights were obtained from vendor catalogues and online sources, assuming accurate representation of real use; OR and PR energy estimates were based on hospital facility data and assumed proportional allocation per surgery duration. Standardized surgical instrument trays; Fixed waste disposal emissions | Stated: Small sample size, only 14 patients had surgeries in both OR and PR settings, limiting generalizability. Hospital-specific data; Limited scope of carbon footprint: The analysis only considers immediate surgical emissions, excluding long-term environmental impacts.  Not stated: No differentiation of material recyclability; No assessment of alternative anaesthesia methods: While TIVA is assumed to be low-impact, comparisons with regional anaesthesia are not explored. |
| **Karam (2024)** | Patient's travel (return trip),Pre‐ and post‐operative consultations, staff commute to healthcare facility, Manufacturing processes and infrastructure for medical devices, raw material transport, Environmental impact embedded in equipment and construction, Manufacturing process of active pharmaceutical ingredients, pen marking, knee splinting, waste generated by implants | Energy consumption per surgery estimated from hospital benchmarks. Surgical material use estimated from literature and procedural guidelines. Waste management emissions modelled using standard disposal pathways. Waste management emissions modelled using standard disposal pathways. Graft preparation emissions estimated from LCA databases. Allocation factor for reusable instruments. Sterilization process emissions assumed constant, based on literature values. | Stated: no real-time data collection or operating room audits, study conducted in a single hospital, no structured uncertainty analysis.  Not Stated: no detailed assessment of supply chain emissions, no clear type of data evaluation, no consideration of alternative waste treatment methods (e.g., recycling or waste-to-energy). |
| **Kodumuri (2024)** | Manufacturing and supply chain emissions of implants and surgical materials, transportation emissions for implants and surgical supplies, pharmaceutical | Assumption on energy consumption (based on 210m³ theatre space for 90 minutes per case), sterilization energy calculation (based on two cycles per hip tray and divided among case), reusable impact divided by 2,040 uses (a commonly used LCA assumption). | Stated: Exclusion of Scope 3, the actual CF of implant production is likely much higher than what is reported, results apply to only one hospital in the UK, making it difficult to generalize to other healthcare systems. Not stated: The study claims to exclude scope 3, but the CF of all materials contains scope 3, not scope 1 which is associated to direct emissions (ex volatile anaesthesia).  Not stated: Carbon footprint calculations assume a standardized THA procedure but do not account for variations in surgical time, patient complexity, or unexpected complications. |
| **Mousania (2024)** | HVAC, capital equipment, manufacturing of multi-use instruments, patient and staff transportation to and from the hospital are not included; emissions and energy of the manufacturing phase itself (gate); Recycling, especially for plastic and cardboard packaging it is ignored in this study | Electricity consumption per device is assumed from technical specifications; Plastic and metal waste is categorized based on weight and disposal method, Sterilization cycle assumed to be constant; Composition material of instruments assumed to be constant; A 20-year lifespan was assigned to multi-use instruments projecting 5000 uses before End of Life; one decontamination process is completed for one ESS; one manufacture and one distribution site for all materials. | Stated: May underestimate the total carbon footprint of reusable surgical instruments and sterilization processes. Assumes either landfill or incineration, without considering regional variations. Some hospitals may have better recycling or waste-to-energy systems, affecting overall impact.  Not stated: Lack of primary data collection in the operating room, no real-time audit to measure actual material use, waste, or energy consumption. Study relies on hospital inventory records and literature-based estimates, potentially missing inefficiencies. No assessment of hospital-specific waste management efficiency, which could alter the carbon footprint results. |
| **Nakarai (2024)** | Manufacturing phase of each product, disposal of surgical implants and bone-grafts products, waste treatment variability, transportation details | Fixed formulas for sterilization process (electricity consumption and number of uses) and for the quantity of anaesthetic used; assumption on energy consumption in OR theatre | Stated: exclusion of manufacturing phase, which may lead to an underestimation of the total environmental impact; the results are specific to one hospital in New York, USA, and may not be generalizable to hospitals in different countries or regions; limited patient sample (only 15 pairs of patients) were included in the analysis after propensity-score matching; not direct real-time hospital measurements for energy consumption.  Not stated: the reliance on Ecoinvent and Umberto software means that actual CF values may differ from real-world hospital measurements; the study discusses reducing CF by minimizing disposable items and anaesthesia time, but does not assess the potential impact on patient safety and clinical outcomes; the study does not propose specific strategies for sustainable product design, such as biodegradable disposables or alternative sterilization methods. |
| **Parker (2024)** | HVAC; lighting | Laundry energy consumption estimates from literature, plastic waste emissions factor applied universally, patient setup standardization | Stated: The impact of recycling was not included. Not stated: The carbon footprint is not complete because only few factors were included, but results functional for the comparison of the two protocols applied |
| **Shah (2024)** | Waste created by anaesthetic procedure, recycling and laundering process, energy consumption | Waste weight average from 5 procedures pr hospital per surgery type, standardized waste management assumptions based on contractor data, assumes similar material distribution | Stated: small sample size (5 ACLR & 5 RCR per hospital), variability in disposal costs and methods among hospitals, no differentiation of plastic types (recyclable vs. non-recyclable), only waste-related CO₂ emissions are considered, excluding transport & production.  Not stated: no assessment of surgical equipment impact (electronic tools, implants), no quantitative evaluation of waste reduction strategies. |
| **Spil (2024)** | hospital infrastructure, medical equipment manufacturing, staff, patient, and visitor travel, disposal emissions from the placenta, oxytocin, syntometrine, and lubricant gel, linen and furniture | The amount of disposables, PPE, and instruments was assumed based on audit observations, expert review, and national guidelines; average analgesia duration ;i instruments were assumed to undergo 50 reuses before disposal, with a sensitivity analysis for 100, 200, and 300 uses; length of stay was taken from national maternity statistics, with 1.8 days for caesarean and 1.13 days for vaginal births; energy use for hospital births was estimated based on total hospital floor area, assuming 10 hours in a birth room and 2 hours in the operating theatre for caesarean births. | Stated: the study models "ideal" births with no deviations or emergency interventions, which may not fully represent real-world variability; The impact of home-to-hospital transfers and return trips was not analysed; hospital ward energy data may not be generalizable.  Not stated: no assessment of alternative pain relief options; no breakdown of birth-related hospital resource consumption; limited generalizability to other healthcare systems: The study focuses on the UK and the Netherlands, making its findings less applicable to countries with different maternity care models. |
| **Ahmed (2025a)** | Follow-up beyond final dressing clinic due to heterogeneity of any additional procedures performed; international patient travel from calculation (2 patients from Malta excluded from travel bottom-up); hospital infrastructure construction; medications beyond anaesthesia calculated via NHS top-down framework. Functional unit: one patient pathway for multistage autologous microtia reconstruction, from first plastic surgery encounter through final dressing clinic following second stage, including two inpatient admissions. | Hybrid methodology: bottom-up for consultation/operative/dressing-clinic phases, top-down via NHS England Sustainable Care Pathways Guidance (2015) for inpatient bed-days (89.5 kgCO₂e/day assumed as "high-intensity inpatient"); patient travel assumed UK-based petrol car with return journeys x 13 visits across pathway; staff travel from survey, mode x distance per staff type; anaesthesia emissions for stage 1 calculated with sevoflurane + N₂O at 5 L/min, stage 2 with sevoflurane only; sterilization electricity/water per tray x trays per operation; waste weights from stage 2 scaled to stage 1 based on number of suture needles (sharps), swabs (non-infectious offensive), staff (DMR); dressing-clinic emissions limited to patient travel + building energy. | Stated: small sample size (n=23, only 5 completed both stages within study window) affecting generalizability; differing levels of surgical stage completion introduces data variability (pathway fully assessed only in 5/23 patients); retrospective design may not capture current practice nuances; top-down approach for inpatient stay is a rough estimation reducing precision; focus on carbon only-other environmental measures (land/water pollution) not addressed. Not stated: no parameter uncertainty or CI reported; no formal sensitivity analysis; patient safety implications of mitigation strategies (e.g., hub-and-spoke model, reducing in-person consultations) not evaluated; impact of complications excluded; extrapolation to other quaternary plastic surgery procedures limited by specialization. |
| **Ahmed (2025b)** | Inpatient stay (due to highly heterogeneous nature and many day cases at study centre); postoperative follow-up; revisional procedures; future implant exchanges; short-term and long-term complications (e.g., capsular contracture); submuscular vs pre-pectoral differences; hospital infrastructure construction. Functional unit: one patient undergoing immediate unilateral breast implant reconstruction from first appointment regarding implant surgery to leaving recovery area. | Patient/staff travel distances via FreeMapTools with return journeys; patient transport assumed petrol car; staff transport from surveys (mode per staff type); equipment transport via supplier postcodes x weight x freight truck emissions; anaesthesia: TIVA (propofol + remifentanil) - no volatile maintenance gases used in study cohort - calculated per drug dose x operative time; sterilization data from CSU (electricity/water per tray x trays per procedure); one laundry load per procedure at 60 °C; waste weights measured directly for sharps, non-infectious offensive, DMR; scrubs/gowns modeled as single-use per Rizan et al. 2023. | Stated: single-centre retrospective study (n=34); reasonable estimates from existing literature as benchmarks; product-specific carbon data not available from most suppliers (only 1 anaesthetic equipment supplier provided LCA); inpatient stay excluded despite relevance; revisional procedures/implant lifespan (5–8 years per Schrager 2021) not factored; variation between surgeons in preferences overcome via sample size; submuscular vs pre-pectoral differences not explored. Not stated: no parameter uncertainty or CI; no formal sensitivity analysis; capsular contracture (up to 23.8% of patients) and related revisional carbon not quantified; enhanced recovery after surgery protocols impact not assessed; antibiotic prophylaxis carbon impact not assessed; full cradle-to-grave perspective limited by supplier data availability. |
| **Camhi (2025)** | Excluded: staff & patient transportation; hospital cleaning; linen use/reprocessing; food; hospital infrastructure. | Device production modelled via ADEME “Base Carbone” v22.0. Energy assumed from French 2021 electricity mix. Transport assumed: sea freight for intercontinental, truck for land. Reuse lifetimes of instruments based on manufacturer data and literature. | Stated: Device origin sometimes approximated; retrospective equipment list may not capture all variations; results only from two French hospitals; coblation footprint dominated by saline irrigation.  Not stated: No parameter or scenario uncertainty assessed; no sensitivity analysis of device lifetimes or transport assumptions; recycling potential not modelled. |
| **Cohen (2025)** | Excluded: building infrastructure, food, cleaning, long-term equipment; some APIs lacking LCI data (e.g. tranexamic acid); pharmaceutical toxicity beyond CO₂e; upstream supply-chain for certain devices if not traceable. Functional unit: one minimally invasive hospital care pathway (including procedure + stay). | Energy allocation per m² and per patient-day; plug-load averages used for OR equipment when smart-metering unavailable; travel distance assumed by postcode and national modal split; material composition assumed by dominant fraction when not specified; recycling modelled with avoided burden approach; allocation of multi-use instruments based on average lifetimes. | Stated: Results are specific to one Dutch hospital, limiting generalizability; some pharmaceuticals excluded for lack of LCI; scenario analyses dependent on literature EF; large influence of single-use plastics acknowledged but not resolved.  Not stated: No uncertainty quantification for staff/patient travel distances; no sensitivity testing on allocation choices for multi-use instruments; limited assessment of variation between hospitals; exclusions of hospital-wide sustainability interventions (e.g. renewable integration, surgical tray optimization). |
| **Droberz (2025)** | Excluded: sharps waste; waste produced by anaesthesia team; emissions from manufacturing of disposables/reusables; energy use (HVAC, lighting, sterilization); staff and patient travel; recycling process emissions. | Waste weighed per case to nearest 10 g; extrapolation to national caseload based on AOA Joint Replacement Registry and AIHW statistics; assumed constant waste composition per procedure type; used fixed emission factor (0.879 tCO₂e/tonne waste). | Stated: Results limited to intraoperative waste streams; extrapolation assumes homogeneity of practice across hospitals; sharps and anaesthesia waste excluded. Not stated: No uncertainty analysis; no sensitivity testing on emission factors; exclusions of energy, manufacturing and travel likely underestimate total CF; reliance on a single EF may mask variability in waste treatment practices. |
| **John (2025)** | Capital goods used in hospital sterilisation, decontamination units (HSDU), and laundry (LDU),on-site electricity generation, heating, ventilation, and air-conditioning (HVAC). Histology processing emissions were excluded as they were deemed minor. | Energy use: hospital-wide energy consumption was used to estimate TURBT-specific usage, as sub metered readings were unavailable. Operating theatres consume 3-6 times more energy than other hospital areas (based on previous literature). Laundry emissions were calculated using mass-adjusted estimates. Reusable Instrument: sterilisation impact per instrument was calculated based on optimal machine loading. Boiler efficiency was assumed equivalent to the Fulton J-series model. Anaesthesia: sevoflurane use was measured by weighing vaporisers before and after procedures (for five cases). Travel: Patient travel was assumed to be exclusively by car and staff travel emissions were based on a 2021 trust census of 822 staff and 60 patients. Waste Management: Incineration emissions were calculated based on carbon content alone. Recycling emissions were considered out of scope, following GHG protocol guidelines. | Stated Limitations: limited generalizability: results are specific to one UK hospital, limiting applicability to other regions or healthcare systems. Pharmaceutical emissions were modelled using industry-wide averages, rather than drug-specific LCA data. Cooling emissions were omitted, potentially underestimating energy use in operating theatres. Comparison with alternative TURBT techniques (e.g., flexible cystoscopy and laser ablation) was not included. No assessment of the potential impact on patient safety when reducing disposable equipment usage.  Not stated: upstream and downstream supply chain emissions for medical devices were estimated but not comprehensively assessed. No measurement of operative time or variations in patient-specific treatment decisions. While the study suggests switching to reusable drapes and limiting irrigation, a full LCA comparison of these options is not provided. |
| **Mattei (2025)** | Staff transportation, hospitalization, cleaning of OR, reprocessing of linen, water consumption | Each patient assumed petrol car, 5 round trips (Google Maps distance ×10). Average values for OR electricity & heating consumption. Sterilization data normalized by # uses. Carebone database used for medical devices, consumables, pharmaceuticals | Stated: Retrospective data collection; study limited to one hospital (Lille, France); approximations for transport & energy use. Not stated: No parameter or scenario uncertainty analysis; no sensitivity analysis on alternative transport modes or energy mixes. |
| **Silva de Souza L.C. (2025)** | Excluded: manufacturing of OR equipment; anaesthetic gases; some minor items (hydrogel, wooden swabs, implant packaging); building infrastructure. | Assumed electricity mix of Pennsylvania grid; HVAC modelled via Campion bin approach; sterilization and laundry cycles standardized based on manufacturer data; waste disposal assumed 26% landfill, 74% RMW; material composition simplified to dominant component. | Stated: Results partly based on modelled data rather than direct metering; manufacturing phase not included; allocation of laundry transport (350 km) assumed uniform; scenario analysis dependent on literature emission factors. Not stated: No formal uncertainty or sensitivity analysis; no variability ranges for emission factors; exclusions may underestimate total CF |
| **van Bree (2025)** | Excluded: hospital infrastructure, consumed food, room cleaning; combined valve/CABG and off-pump CABG; toxicity of pharmaceuticals beyond CO₂e; long-term equipment outside procedure | OR HVAC modelled with 68 air changes/h; electricity mix from Dutch national DB; commuting distance based on average 23 km; pharmaceuticals: only active ingredient production + packaging; disposables assumed single-material dominated; waste disposal split 26% landfill, 74% RMW; laundry transport standardized. | Stated: Some pharmaceuticals excluded (toxicity impacts); assumptions for material types when unavailable; generalizability limited to Dutch academic hospitals; staff commute methodology debated in LCA standards. Not stated: No detailed allocation of multi-use equipment lifetimes; uncertainty in emission factors not fully explored for all categories; no patient-reported variability in travel; exclusion of infection-prevention related cleaning may underestimate footprint. |
